# Tobacco Smoke Activates Human Papillomavirus 16 p97 Promoter and Cooperates with High-Risk E6/E7 for Oxidative DNA Damage in Lung Cells

**DOI:** 10.1371/journal.pone.0123029

**Published:** 2015-04-01

**Authors:** Nelson Peña, Diego Carrillo, Juan P. Muñoz, Jonás Chnaiderman, Ulises Urzúa, Oscar León, Maria L. Tornesello, Alejandro H. Corvalán, Ricardo Soto-Rifo, Francisco Aguayo

**Affiliations:** 1 Virology Program, Institute of Biomedical Sciences, Faculty of Medicine, University of Chile, Santiago, Chile; 2 Cellular and Molecular Biology Program, Institute of Biomedical Sciences, Faculty of Medicine, University of Chile, Santiago, Chile; 3 Molecular Biology and Viral Oncology Unit, Istituto Nazionale Tumori "Fondazione G. Pascale"—IRCCS, Naples, Italy; 4 Advanced Center for Chronic Diseases (ACCDiS) and UC—Center for Investigational Oncology (CITO), Pontificia Universidad Católica de Chile, Santiago, Chile; Georgetown University, UNITED STATES

## Abstract

We have previously shown a functional interaction between human papillomavirus type 16 (HPV-16) E6 and E7 oncoproteins and cigarette smoke condensate (CSC) in lung cells suggesting cooperation during carcinogenesis. The molecular mechanisms of such interaction, however, remain to be elucidated. Here we first present evidence showing that cigarette smoke condensate (CSC) has the ability to activate the HPV-16 p97 promoter by acting on the long control region (LCR) in lung epithelial cells. Interestingly, we observed that CSC-induced p97 promoter activation occurs in a dose-dependent manner in both tumor A-549 (lung adenocarcinoma), H-2170 (bronchial carcinoma), SiHa or Hela (cervical carcinoma) cells but not in non-tumor BEAS-2B (bronchial) or NL-20 (alveolar) lung cells unless they ectopically expressed the HPV-16 E6 and E7 oncogenes. In addition, we also observed a significant increase of primary DNA damage in tumor and non-tumor CSC-treated lung cells expressing HPV-16 E6 and E7 oncogenes suggesting a cooperative effect in this process, even though the contribution of E7 was significantly higher. Taken together, our results strongly suggest that tobacco smoke is able to induce the activation of the HPV-16 p97 promoter in cooperation with HPV-16 E6 and E7 oncogenes that, in turn, sensitize lung cells to tobacco smoke-induced DNA damage.

## Introduction

Lung cancer is a leading cause of cancer-related death in the world [1]. Although tobacco smoking plays a major role in the development of this disease, other factors are also relevant towards its development [2]. Human papillomavirus (HPV) has been detected in primary lung carcinomas with frequent integration into the host genome, suggesting a causal association in a subset of subjects [3].

However, the presence of HPV is highly variable in different geographic regions. In 2012, a meta-analysis concluded that HPV is present in 22% of lung carcinomas worldwide [4]. Specifically, the HPV-16 genotype, the most frequent HPV type in cervical carcinomas, has been found in a subset of lung carcinomas [3,5]. Moreover, E6 and E7 transcripts have been detected in HPV positive lung tumors, suggesting a functional viral activity [6]. However, other studies failed to detect the expression of these oncogenes, even though HPV was detected in a subset of cases [7] and therefore the involvement of HPV in lung carcinogenesis has not been clearly elucidated yet. Studies in Asia reported a role for HPV in lung cancer as a smoke cigarette independent carcinogen due to its presence in lung adenocarcinomas from non-smoking women [8]. On the other hand, an international pooled analysis reported that among the HPV positive lung carcinomas, 71% belong to smoking or former smoking groups [9]. Thus, the potential involvement of HPV in lung cancer associated with tobacco smoke is a concern that warrants further investigations. We have previously reported that HPV-16 E6 and E7 oncoproteins and cigarette smoke condensate (CSC) cooperate increasing the proliferative and tumor properties of lung epithelial cells [10]. Although the mechanism by which tobacco smoke and HPV are able to interact in lung cells is unknown, it is widely accepted that constitutive high-risk (HR)-HPV E6 and E7 expression is necessary for cell immortalization and for the maintenance of the tumor phenotype [11]. However, HPV-immortalized cells are not tumorigenic in animal models suggesting that additional molecular alterations are necessary for complete HPV-induced tumoral transformation [11].

The HPV genome is organized in three regions: early, late and the long control region (LCR). The LCR is a non-coding region that spans approximately one thousand nucleotides and plays critical roles in the regulation of viral gene expression [12]. The HPV early promoter controls the transcription of all early genes and among them the E6 and E7 oncogenes are expressed as a polycistronic transcript [13]. The AP-1 and Yin-yang-1 (YY1) transcription factors are known to induce activation and repression of the early promoter through their binding to specific sites into the LCR [14,15]. On the other hand, the viral E2 protein is able to bind to E2 binding sites (E2BSs) located downstream of the LCR, repressing gene expression directed by the HPV early promoter [16]. However, E2 expression is lost following the HPV integration into the host genome leading to E6 and E7 overexpression and the consequent destabilization of p53 and pRb tumor suppressor proteins, respectively [12].

Tobacco smoke is a complex mixture of more than 4 000 compounds and more than sixty of them have a demonstrated carcinogenicity [17]. Previously, it has been reported that benzo[a]pyrene, a polycyclic aromatic hydrocarbon present in tobacco smoke, is able to increase the expression of the E7 oncoprotein in cervical cancer cells [18]. In addition, Alam et al. demonstrated that benzo[a]pyrene is able to increase the number of HPV genomes or virions depending on its concentration in human cervical keratinocytes [19]. Moreover, the MAPK/ERK pathway was activated after benzo[a]pyrene incubation [20]. On the other hand, it has been reported that nicotine, a component of tobacco smoke, is able to activate the LCR from high risk-HPV in human cervical keratinocytes in collaboration with the cellular transcription factor Brn-3a [21]. Since the upper respiratory tract and aero-digestive epithelia are potentially exposed to both HPV and environmental contaminants such as tobacco smoke, an interaction between both carcinogenic agents is plausible [10]. In this study, we showed that tobacco smoke was able to activate the HPV-16 p97 promoter in a dose dependent-manner in tumor lung cells when it is under the control of its cognate LCR. In non-tumor lung cells such activation occur exclusively upon HPV-16 E6/E7 oncoproteins expression that, in turn, were able to sensitize tumor and non-tumor lung cells for tobacco smoke-induced DNA damage. This study suggests new insights about the interaction between HPV and tobacco smoke in cancer development.

## Materials and Methods

### Cell lines and culture media

A-549 (CCL-185), BEAS-2B (CRL-9609), H-2170 (CRL-5928), NL-20 (CRL-2503), HeLa (CCL-2) and SiHa (HTB-35) cell lines were obtained from American Type culture collection (ATCC) (Manassas, VA). A list with the features of each cell line used in this study is shown in S1 Table. The A-549, BEAS-2B, H-2170, HeLa and SiHa cells were incubated in RPMI1640 (Invitrogen) basal medium supplemented with 10% fetal bovine serum (FBS) (Hyclone) and antibiotics (gentamicin, penicillin and streptomicin) and maintained at 37°C with 5% CO2 atmosphere. The NL-20 (CRL-2503) cells were maintained in BEBM basal medium more supplements according the conditions established by the manufacturer (Lonza Inc.). For subculture, the cells were incubated with trypsin for 3–5 min and maintained with new medium containing FBS. The cells were periodically tested for mycoplasma contamination.

### DNA cloning

The primers for LCR/p97 amplification (1020 bp) were designed using the GenBank HPV-16 isolate QV33501. The primers used in this study are shown in S2 Table. The software ApE-A plasmid editor was used. A reporter system was developed using the expression vector pmiR-GLO (Promega). The native PGK promoter was deleted by BglII and ApaI digestion and the HPV-16 LCR/p97 region amplified by PCR (1020 bp) from SiHa cells was inserted upstream of the firefly luciferase reporter gene (luc2). Renilla luciferase present in the vector was used to normalize the firefly luciferase activity. In addition, the p97 promoter region was amplified from SiHa cells and was inserted into the pmiR-GLO vector (Promega) as indicated above.

### Stable and transient transfections

Transient transfections of the pmiR-GLO construct containing the HPV-16 LCR/p97 were carried out in A-549 (lung adenocarcinoma), H-2170 (lung tumor bronchial), NL-20 (non-tumor alveolar), NL-20/E6E7 (ectopically expressing HPV-16 E6/E7), BEAS-2B (non-tumor bronchial), BEAS-2B/E6E7 (ectopically expressing HPV-16 E6/E7), HeLa (HPV-18 positive cervical cancer) and SiHa (HPV-16 positive cervical cancer) cells. Briefly, the cells were incubated with the corresponding culture medium until they reach to 80% confluence and then transfected with Lipofectamin 2000 (Invitrogen) using 500 ng of recombinant vector per well. The cells were then incubated for 6 hours at 37°C in an incubator with 5% CO2 atmosphere. After, the supernatant was discarded; the cells were washed with phosphate buffered saline (PBS) and incubated for 16 hours with different concentrations of CSC (Kentucky University) in the corresponding culture medium. NL-20 and BEAS-2B cells were previously stably transfected with pLXSN or pLXSNE6E7 constructs (kindly donated by Massimo Tommasino, IARC, France) according to previously published procedures and were maintained in RPMI1640 medium containing 10% FBS, antibiotics and 0.1 mg/mL G418 [10].

### Luciferase assays

The cells transfected with the pmiR-GLO plasmid were exposed to concentrations of CSC ranging 0.1 to 50 μg/mL (Kentucky University) and measurements of luciferase activity were made after 16 hours of incubation as previously described [22]. The Kit Dual-Luciferase® Reporter Assay System (Promega) was used. The cells were depleted of culture medium and were washed with 400 μL of PBS. Then 75 μL of passive lysis buffer (Promega) were added to each well and were incubated for 1 hour at room temperature. Next, a lysis through 15 spins down with the micropipette was performed and twenty microliters of cell extracts were added to each well in a 96 wells-microplate. The measurements were made in a GLOMAX96 luminometer 1.9 (Promega) as recommended by the manufacturer. Finally, the Firefly luciferase activity was normalized against the activity of Renilla luciferase and presented as percentage of the control.

### Tobacco smoke condensate and hydrogen peroxide expositions

The A-549 and BEAS-2B cells were cultured until 80% of confluence was reached followed by serum depletion for 24 h. The cells were exposed to CSC (Kentucky University) dissolved in RPMI-1640 medium at different dilutions (1 to 50 μg/mL) in DMSO. Cells exposed to DMSO were included as controls for each experiment. In addition, the cells were exposed to hydrogen peroxide at a concentration of 100 μM for A-549 cells and 10 μM for BEAS-2B cells. A toxicity curve was previously made for each cell line. The hydrogen peroxide exposition was made for a period ranging from 5 to 60 min on ice.

### Single cell electrophoresis (Comet Assay)

The single cell electrophoresis (Alkaline Comet Assay) (Trevigen) assay was carried out in bronchial BEAS-2B and A-549 cells ectopically expressing HPV-16 E6/E7 oncogenes that were also exposed to different concentrations ranging from 1 to 50 μg/mL CSC (Kentucky University) and times ranging from 24 to 96 h at 37°C. Single cell electrophoresis (Comet assay) (Trevigen, Gaithersburg) was made according to the manufacturer instructions. Briefly, A-549 or BEAS-2B cells were incubated with CSC (Kentucky University) or hydrogen peroxide (Merck) as previously described [10]. The cells were trypsinized and washed two times with PBS and were suspended at 10^5^ cell/mL. Then, cells were mixed with 1% low melting point agarose (Winkler) at 37°C with a volume relationship cells/agarose of 1:10. Fifty microliters of cells/agar mixture was added to a comet-slide for 10 min at 4°C. The slides were incubated in lysis solution on ice for 45 min and immersed in alkaline solution pH 13 for 40 min at room temperature in the darkness. Afterwards, the slides were electrophoresed at 21volts for 30 min in alkaline buffer. The slides were then washed twice with distilled water for 5 min, immersed in 70% ethanol for 5 min and dried for 15 min at 45°C. One hundred microliters of SYBR Green (Trevigen) were added to the agarose and were stored at 4°C for 10 min in the darkness. The comets were visualized using a fluorescence microscope (Nikon, Microphot-FXA), photographed and the total area of each comet was measured using the CometAssay IV^TM^ software. Fifty comets were measured per treatment.

### RNA purification, cDNA preparation and reverse transcriptase quantitative PCR (RT-qPCR)

Total RNA was isolated using 1 mL of Trizol reagent (Invitrogen) according to the manufacturer’s instructions. The RNA concentration and purity were determined using a Nanodrop1000 spectrophotometer (Thermo Scientific). The integrity of the RNA was determined by 0.9% agarose gel electrophoresis. One microgram of RNA were treated with RQ1 DNAse (Promega) at 37°C for 1 hour and denatured at 65°C. The cDNA was prepared in a 20 μL-reaction volume containing DNAse-treated RNA, 1 U RNase inhibitor (Promega), 0.04 mg/mL random primers (Promega), 2 mM dNTP (Promega) and 10 U Moloney murine leukemia virus (MMLV) reverse transcriptase (Promega). The reaction mixture was incubated for 1 h at 37°C followed by incubation at 70°C for 10 min. The cDNA samples were subjected to PCR amplification using specific primers for HPV-16 E6 and GAPDH (S2 Table). For GAPDH, 1 μL of the prepared cDNA was added to a tube containing 1X reaction buffer; SENSI-MIX 2X (Bioline); 0.52 mM primer pairs and nuclease-free water. PCR amplification was performed under the following conditions: denaturation at 94°C for 5 min followed by 35 cycles of 94°C for 30 s, 55°C for 30 s, 72°C for 30 s and final extension for 5 min at 72°C. For E7, 1 μL of the prepared cDNA was added to a tube containing 1X reaction buffer; SENSI-MIX 2X (Bioline); 0.52 mM primer pairs and nuclease-free water. PCR amplification was performed under the following conditions: denaturation at 95°C for 5 min, followed by 35 cycles at 95°C for 1 min, 55°C for 1 min, 72°C for 1 min and final extension for 10 min at 72°C. Amplified products were characterized by melting analysis and using 3% agarose gel electrophoresis, stained with MasterSafe (MaestroGene) and observed with a UV-transilluminator (Vilber-Lourmat).

### Immunofluorescence for γH2AX phosphorylation

After HPV-16 E6 and E7 transfections and CSC-treatments, the cells were seeded, incubated overnight in coverslips and the culture medium was discarded. Then the cells were washed for 5 min in PBS. The coverslips were dried and incubated with cold acetone (previously frozen at -20°C) and were incubated for 5 min at room temperature. For blocking, the coverslips were incubated with 5% bovine seroalbumin (BSA) for 30 min at room temperature. The primary antibody was incubated in diluted 1/100 in PBS-5% BSA for 1 hour. Three washes with PBS were carried out lasting 6 min at room temperature. The secondary antibody was FITC-labeled and diluted 1/30 in PBS-5% BSA and incubated for 1 hour under a dark room. Three washes were made and the coverslips were mounted with glycerol and the reading was made in a fluorescence microscope (Nikon, Microphot-FXA).

### Lentiviral transduction of shRNA for E6/E7 silencing

Five previously described hairpins designed to knockdown HPV-16 E6/E7 expressions were cloned in the pLKO.1 plasmid (Thermo Scientific), into the AgeI and EcoRI sites. Oligonucleotides including overhangs, hairpin sequences and transcription terminators were hybridized and ligated into the plasmid. Selected and sequence verified constructs were co-transfected in 293T cells with helper plasmids to obtain lentivirus particles. After testing its efficiency by RT-qPCR of E7 mRNA, we selected target shHPV186 [23] for testing in the Comet Assay using a scramble hairpin as control.

### Statistical analysis

The data obtained using comet assay were normalized using Shapiro-Wilk test and the comparison of averages was made using Student-t test with Stata 11 software. P-values less than 0.05 were considered statistically significant.

## Results

### Cigarette smoke condensate activates the HPV-16 LCR/p97 promoter in a dose-dependent manner in lung and cervical cancer cells

**Fig 1:**
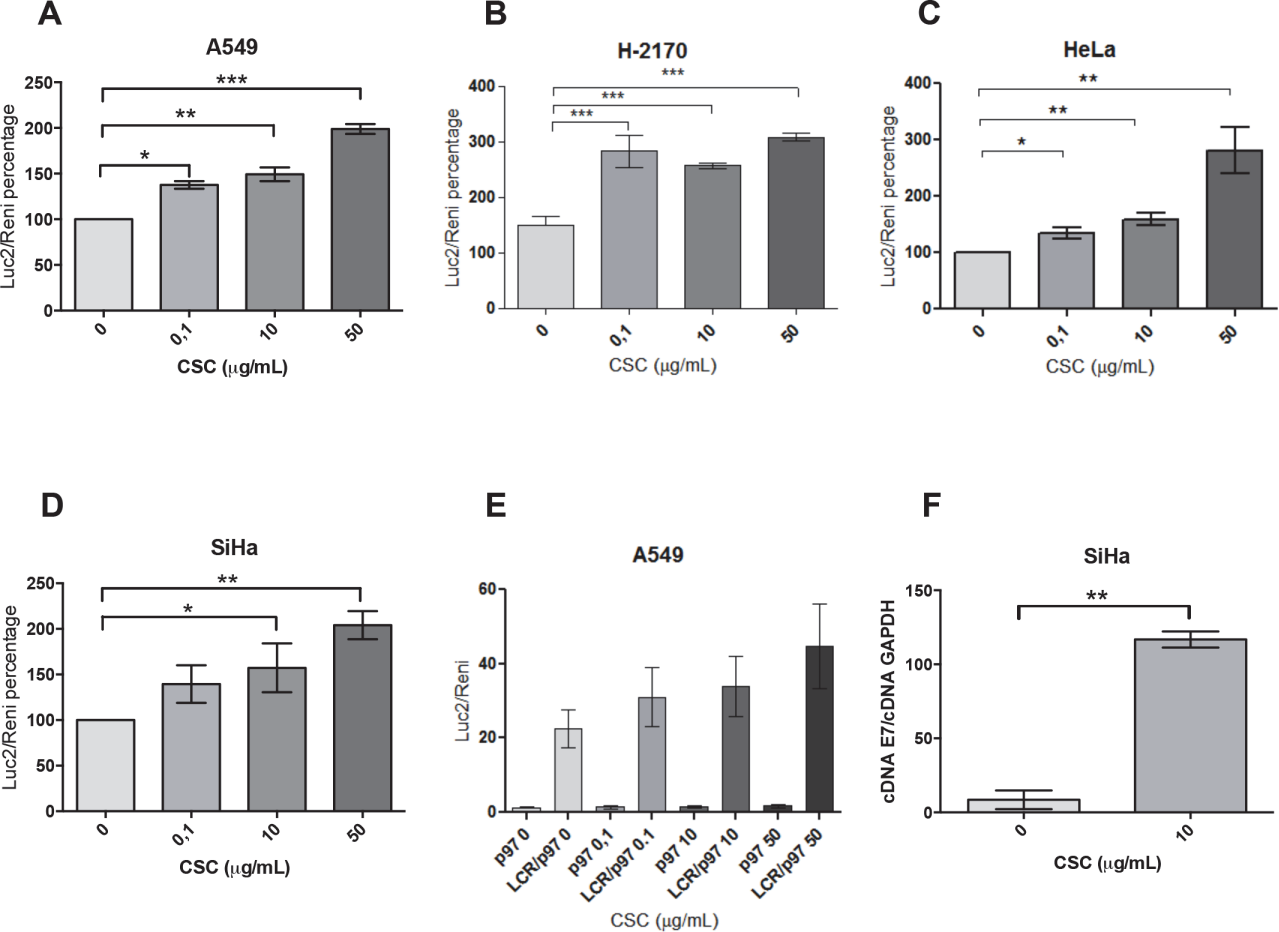
Cigarette smoke condensate (CSC) activates the HPV-16 p97 promoter in a dose-dependent manner in lung and cervical cancer cells. (**A**) A-549 (**B**) H-2170 (**C**) HeLa and (**D**) SiHa cells were exposed to CSC (0.1–50 μg/mL) for 16 hours after transfection with the pmiR-GLO construct containing the complete HPV-16 LCR/p97 region. (**E**) A-549 cells were transfected with the pmiR-GLO containing only the HPV-16 p97 promoter and the luciferase activity was compared with those cells transfected with the full HPV-16 LCR/p97 region. (**F**) SiHa cells were exposed to 10 μg/mL CSC and the levels of E7 transcripts were measured using RT-qPCR. The graphs are representative of three independent experiments. (* = p<0.05; ** = p<0.01; *** = p<0.001, Luc2/Reni percentage ± SEM).

The full-length HPV-16 LCR and p97 promoter region were amplified by PCR from DNA extracted from SiHa cells and inserted upstream the luc2 reporter gene into the pmiR-GLO vector (S1 Fig). This construct also harbors the Renilla luciferase reporter gene under the control of the SV40 promoter, which was used as an internal control. We used this construct to analyze the activity of the p97 promoter in the context of HPV-16 LCR in epithelial lung and cervix-uterine cancer cells exposed to different concentrations of CSC. Previously, toxicity assays were carried out in cell lines used in this study (S2 Fig). Concentrations that not induce a significant cell death were used. In addition, these CSC concentrations were in the range of physiological interest [10]. We observed that CSC was able to activate the HPV-16 p97 promoter in a dose-dependent manner (0.1–50 μg/mL) in A-549, H-2170, HeLa and SiHa tumor cells. In fact, a statistically significant difference respect to the control was observed when A-549, H-2170 and HeLa cells were exposed to 0.1 (p<0.05), 10 (p<0.01) and 50 (p<0.001) μg/mL CSC (Fig 1A–1C). We observed the same response in SiHa cells (cervix uterine cancer cells harboring 1–2 copies HPV-16/cell) exposed to 10 (p<0.05) and 50 (p<0.01) μg/mL CSC (Fig 1D). Then, we went on to determine the p97 promoter activity in the absence of an intact HPV-16 LCR. For this, we created a pmiR-GLO construct containing only the p97 promoter that was used to determine the luciferase activity in A-549 lung cells exposed to CSC. As expected, we observed that p97 promoter activity was significantly lower compared to those cells transfected with the full HPV-16 LCR/p97 construct confirming the critical role of the LCR in p97 promoter activation (Fig 1E). Moreover, CSC was not able to activate the p97 promoter in the absence of the LCR suggesting that transcriptional activation occurs at the LCR level. In order to confirm that p97 activation by CSC leads to an increase in the level of the E6E7 transcript, we exposed HPV-16 positive SiHa cells to 10 μg/mL CSC and quantified the E7 transcript by RT-qPCR. We observed a significant increase in the viral transcript as showed in (Fig 1F). Thus, these results suggest that p97 promoter is activated by CSC in the presence of a full HPV-16 LCR in the context of lung or cervix-uterine cancer cells.

### CSC-mediated activation of the HPV-16 LCR/p97 promoter in non-tumor lung cells occurs in the presence of HPV-16 E6 and E7 oncogenes

**Fig 2:**
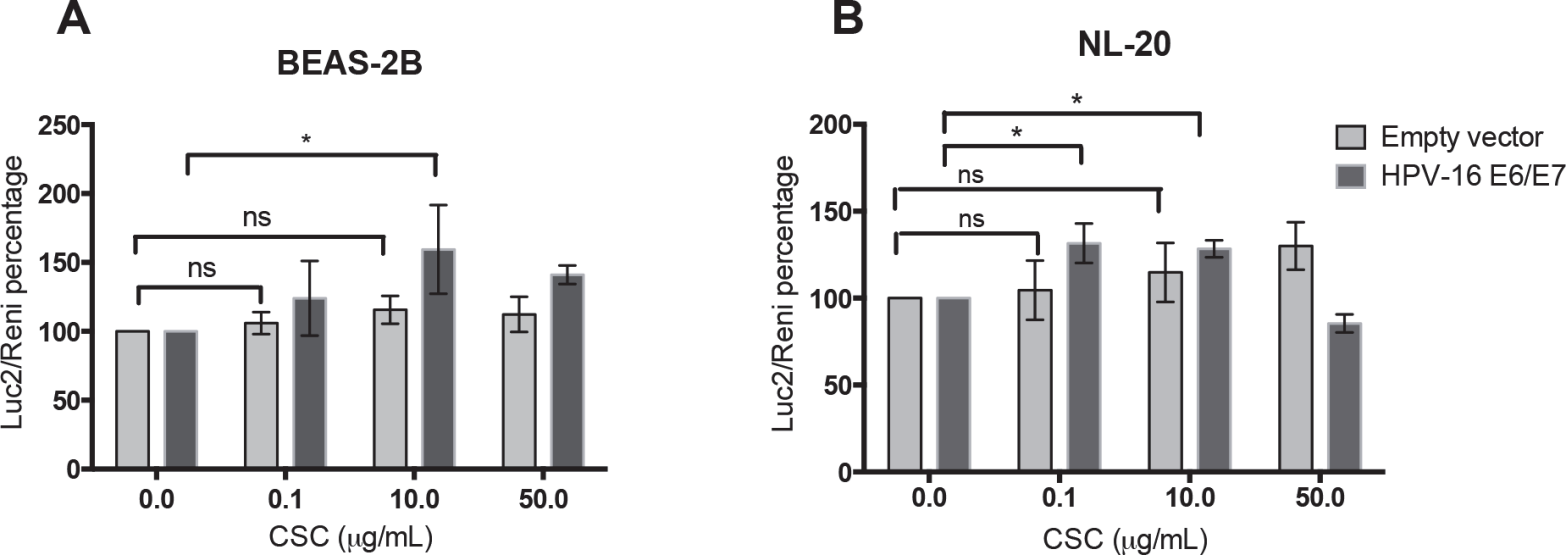
Cigarette smoke condensate (CSC) activates the HPV-16 p97 promoter in a dose-dependent manner in non-tumor lung cells after HPV-16 E6/E7 expression. (A) BEAS-2B, (B) NL-20 cells stably transfected with HPV-16 E6/E7 oncogenes (dark) or an empty vector (gray) were transfected with the pmiR-GLO containing the full HPV-16 LCR/p97 region. The graphs are representative of three independent experiments. (* = p<0.05; ** = p<0.01; *** = p<0.001, Luc2/Reni percentage ± SEM).

We evaluated the HPV-16 p97 promoter activity in the context of non-tumor BEAS-2B and NL-20 lung cells. The experiments performed as above revealed that the p97 promoter was not activated when they were exposed to CSC at concentrations ranging from 0 to 50 μg/mL (Fig 2A and 2B, gray bars). Previously it was reported that HPV-16 E6 oncoprotein has the ability to induce the expression of the AP-1 heterodimer component c-fos, a known transcription factor involved in p97 promoter activation [24,25]. Thus, we analyzed the activity of the p97 promoter in non-tumor lung cells in the presence of ectopic expression of HPV-16 E6 and E7 oncogenes. For this, we took advantage of the previously described BEAS-2B/E6E7 cell lines, which constitutively express functional HPV-16 E6 and E7 oncoproteins [10] to evaluate p97 promoter activity in response to CSC exposure. In BEAS-2B/E6E7 cells we found a significant increase of luciferase activity in the presence of 10 μg/mL CSC (p<0.05)(Fig 2A, dark bars). In NL-20/E6E7 cells we found a significant increase of luciferase activity in the presence of 0.1 and 10 μg/mL CSC (p<0.05), (Fig 2B, dark bars) suggesting that HPV-16 LCR-driven p97 activation mediated by CSC is influenced by the presence of E6 and E7 viral oncoproteins.

### HPV-16 E6 and E7 oncogenes sensitize lung cells to tobacco smoke-induced DNA damage

**Fig 3:**
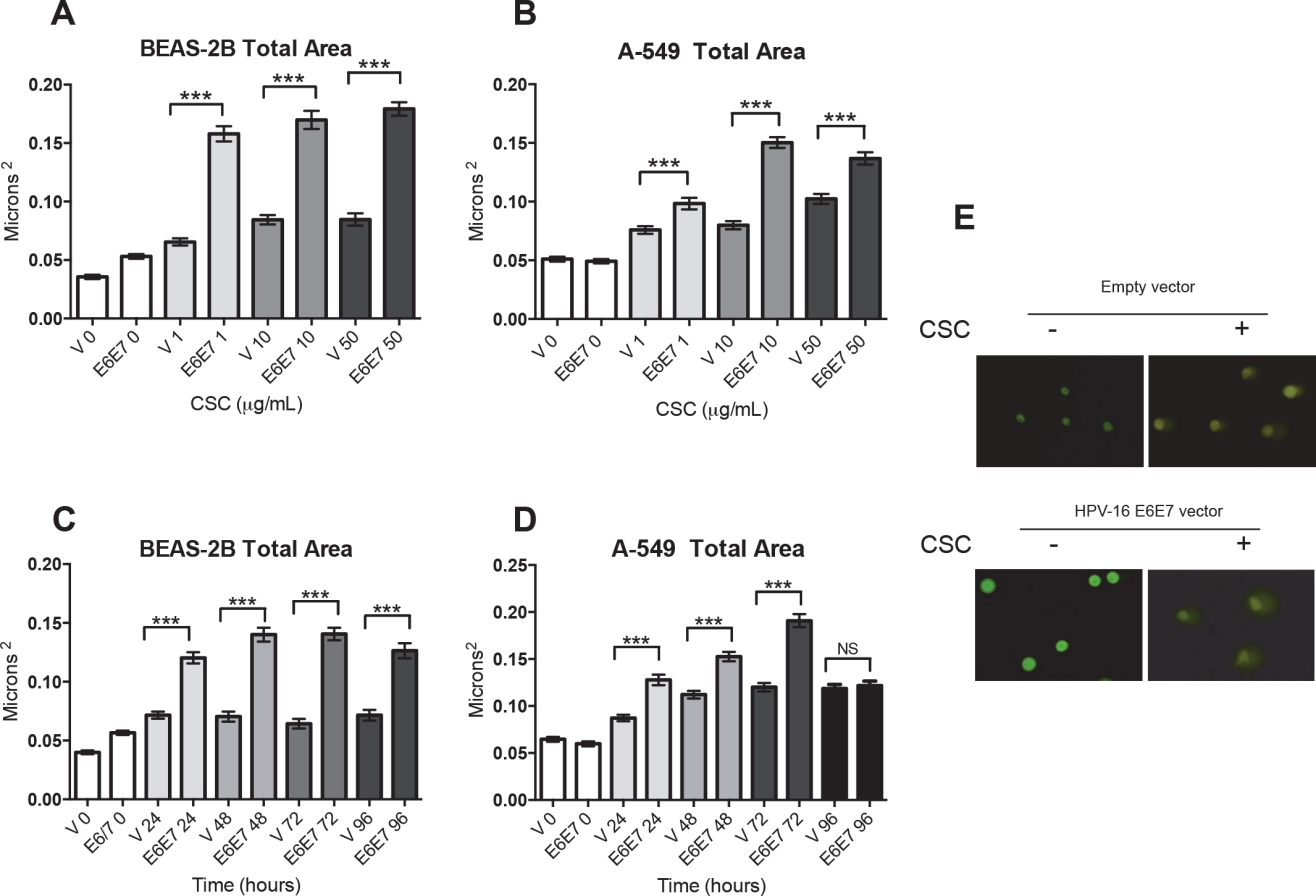
HPV-E6/E7 oncogenes sensitize lung cells for tobacco smoke-induced DNA damage. (A) BEAS-2B and (B) A-549 cells were exposed for 72 hours to 1–50 μg/mL CSC and analyzed using comet assay. (**C**) BEAS-2B and (D) A-549 cells were exposed to 10 μg/mL CSC for 0–96 hours. (E) Image showing comets in A-549 cells transfected with HPV-16 E6E7 or an empty vector and exposed to 10 μg/mL CSC. The graphs are representative of three independent experiments (* = p<0.05; ** = p<0.01; *** = p<0.001, Total Area ± SEM).

It has been widely established that tobacco smoke induces oxidative DNA damage in lung cells [17]. Thus, we were interested in to evaluate the DNA damage in the presence of both tobacco smoke and HPV E6 and E7 oncogenes in tumor and non-tumor lung cells. For this, both BEAS-2B/E6E7 and A-549/E6E7 cell lines were exposed to different concentrations of CSC and the DNA damage was evaluated using the Comet Assay under alkaline conditions. Compared to the control, we observed a significant increase in primary DNA damage (single and double strand breaks and alkali-labile sites) in HPV-16 E6/E7 expressing lung cells when exposed to CSC ranging from 1 to 50 μg/mL (p<0.001), (Fig 3A and 3B). Then, the lung cells were exposed to 10 μg/mL CSC at different exposition times ranging between 0 to 96 hours. As before, a statistically significant difference was observed in HPV-16 E6/E7-expressing BEAS-2B and A-549 cells incubated with CSC between times ranging from 48 to 96 hours (p<0.001) and 48 to 72 hours (p<0.001) respectively, suggesting that E6 and E7 expression increases CSC-induced DNA damage in lung cells (Fig 3C and 3D). The Fig 3E is a microscopy image of a typical Comet Assay in A-549 cells ectopically expressing HPV-16 E6 and E7 oncogenes and exposed or not to CSC. Next, we also analyzed the effect of hydrogen peroxide, a known oxidative stress agent, on DNA damage using the BEAS-2B and A-549 cell lines described above. As a positive control, the lung BEAS-2B and A-549 cells were exposed to hydrogen peroxide at 10 μM and 100 μM, respectively. We determined the time-dependent response to hydrogen peroxide by analyzing in a time period ranging from 0 to 60 min (S3A and S3B Fig). We observed a significant increase in DNA damage in both lung cell lines exposed to hydrogen peroxide during times ranging 20 to 60 min (p<0.001). Importantly, the effect of hydrogen peroxide was exacerbated in the presence of E6 and E7 oncoproteins suggesting a role of these viral oncoproteins in sensitizing cells to DNA damage. Finally, in order to confirm that DNA damage is occurring in HPV-16 E6 and E7 expressing A-549 and BEAS-2B cells in the presence of CSC, we performed immunofluorescence to detect γH2AX phosphorylation. As shown in S4 Fig, a significant increase in nuclear staining was observed when ectopically expressing HPV-16 E6/E7 BEAS-2B (S4A Fig) and A-549 (S4B Fig) cells were exposed to 10 μg/mL CSC, in comparison with those cells transfected with an empty vector. Taken together, these results strongly suggest that lung cells expressing HPV-16 E6 and E7 oncogenes show an increased DNA damage when exposed to CSC.

### HPV-16 E6 and E7 knockdown using lentiviral transduction of short hairpins RNAs decreases the DNA damage in lung cells

**Fig 4:**
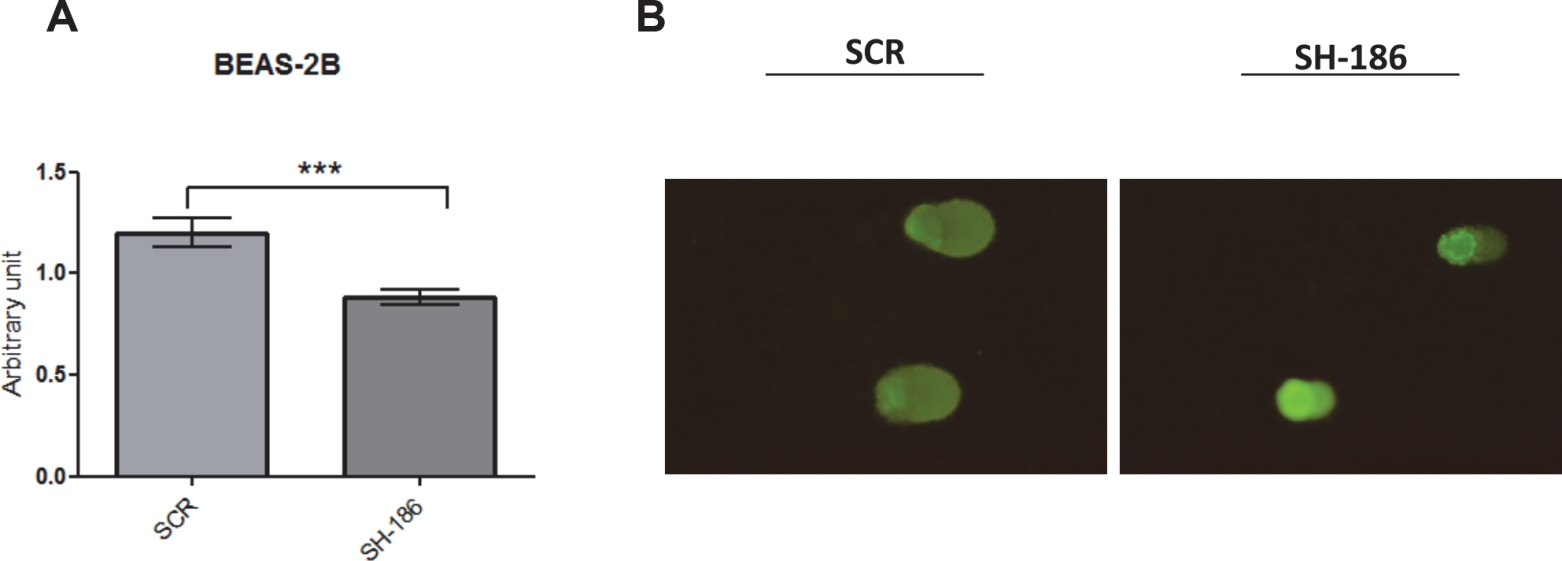
Lentiviral transduction of BEAS-2B cells with an shRNA for HPV-16 E6/E7 silencing reduces the DNA damage induced by CSC exposition. (A) BEAS-2B cells were exposed to 10 μg/mL CSC and incubated with scrambled or shRNA SH-186. The DNA damage was evaluated using comet assay and the total area in each comet was measured. (B) A representative image showing BEAS-2B cells after incubation with SCR or shRNA SH-186.

In order to confirm the role of HPV-16 E6 and E7 oncogenes for inducing DNA damage in cooperation with CSC in lung cells, we silenced E6 and E7 expression by lentiviral transduction of a short hairpin RNA (shRNA). As shown in (Fig 4A), our shRNA was able to decrease significantly the levels of E6 or E7 transcripts in BEAS-2B lung cells that were previously transfected with pLXSNE6E7 vector (S5 Fig). Then, BEAS-2B cells ectopically expressing HPV-16 E6/E7 transcripts were transduced with a lentivirus encoding the E6/E7 shRNA (SH2 o SH186) and exposed to 10 μg/mL CSC for 72 hours. A significant decrease in DNA damage was observed when compared with BEAS-2B cells transduced with lentiviruses encoding for a scrambled RNA sequence (Fig 4B). A representative microphotography of comet assay is shown in Fig 4C. These data suggest that the observed DNA damage in lung cells exposed to CSC is mediated through HPV-16 E6 and/or E7 expression.

### HPV-16 E6 and E7 oncogenes are associated independently with DNA damage in lung cells

**Fig 5:**
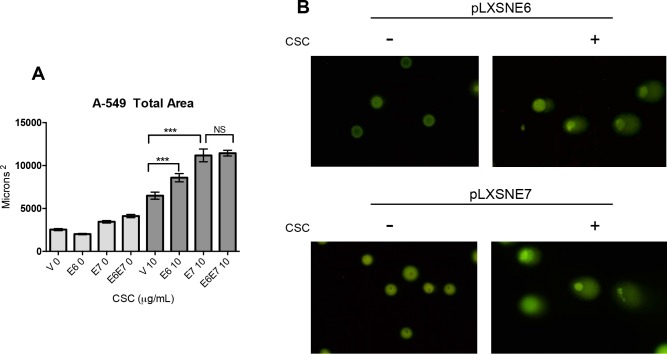
HPV-16 E6 and E7 oncogenes independently are able to sensitize cells to DNA damage. (A) A-549 cells expressing HPV-16 E6 or E7 oncogenes were exposed to CSC at 10 μg/mL and DNA damage was evaluated using Comet Assay. (B) Image showing comets in A-549 cells transfected with pLXSNE6 (upper) or pLXSNE7 (below) in the presence or absence of CSC. (*** = p<0.001, Total Area ± SEM).

We were interested in knowing if some specific HPV-16 oncoprotein or both were involved in the CSC-induced DNA damage in lung cells. Therefore, A-549 cells were stably transfected with a pLXSN vector encoding for either E6 or E7 oncoproteins and exposed to different concentrations of CSC. As shown in Fig 5, E6 and E7 oncoproteins were independently able to sensitize A-549 cells to DNA damage in the presence of 10 μg/mL CSC (p<0.001). However, this effect was significantly higher in HPV-16 E7 transfected cells. HPV-16 E6 oncoprotein was not able to significantly increase the DNA damage caused by HPV-16 E7 oncoprotein alone suggesting that E7 plays a predominant role in CSC-induced DNA damage (Fig 5A and 5B).

## Discussion

Tobacco smoke is the most important cause of lung cancer and others tumors such as a subset of oropharynx, larynx, esophagus and upper airway malignancies. However, tobacco smoking is not a sufficient condition for lung cancer development. In fact, only a low percentage of smokers finally develop this kind of tumor suggesting that additional cofactors are involved in the development of this disease [2].

Early studies demonstrated that HPV-16 and 18 are able to immortalize tracheal and bronchial cells, and even though a commercial HPV positive lung cancer cell line is not available, it was previously reported the establishment of a HPV-16 positive lung cancer cell line from a pleural effusion [26]. Thus, it seems biologically possible that HPV be involved in transformation of lung cells and potentially involved in lung carcinogenesis. In fact, patients with recurrent respiratory papillomatosis infected with HPV-6 or -11 or another HPV genotype have an increased risk of lung cancer [27,28]. However, a very important issue is if HPV presence in lung cancer is epidemiologically relevant and which one is the relationship with other known carcinogens into the lungs such as tobacco smoke. In this respect, HPV infection has been found in lung carcinomas of both smokers and non-smokers in different populations worldwide [4,9]. Recently, an international pooled analysis taking into account Asia, Europe and South/Central America established that 71% of HPV positive lung carcinomas were from smokers. Interestingly, 87% of HPV-positive lung carcinomas in North America were from smokers [9]. Therefore, the notion that the role of HPV when detected in lung cancer could be related to collaborate with tobacco smoke for carcinogenesis is plausible. However, a statistically significant association between HPV and tobacco smoke has not been found. Moreover, previously it has been reported that HPV works as an independent carcinogen for lung adenocarcinoma development in non-smoker women from Taiwan [8].

In a previous work, we reported a functional association between HPV-16 E6/E7 oncogenes and tobacco smoke in lung epithelial cells. In fact, CSC was able to increase the proliferative and tumor properties of lung epithelial cells ectopically expressing HPV-16 E6 and E7 oncogenes [10]. In this study, we present consistent evidence showing that CSC is able to collaborate with HPV in lung cells through at least two different mechanisms. The first is the ability to stimulate the activity of the HPV-16 p97 promoter in the context of an intact HPV-16 LCR (Fig 1 and 2). Interestingly, this activation was only observed in tumor cells such as A-549, H-2170, HeLa or SiHa while non-tumor cells such as BEAS-2B and NL-20 showed activation of p97 promoter only in the presence of ectopic HPV-16 E6/E7 expression. These results suggest that certain properties of tumor cells confer a special susceptibility for p97 activation by CSC in the context of the HPV-16 LCR. Interestingly, HPV-16 E6 and E7 expression seem to resemble such conditions, conducting to p97 promoter activation induced by CSC. As mentioned before, a plethora of transcription factors are upregulated in tumor cells with the possibility to interact with the HPV-16 LCR [13,29]. It is known that regulation of E6 and E7 gene expression is a complex process that involves cellular and/or viral elements or transactivators into the LCR conducting the activation of p97 promoter in HPV-16, p99 in HPV-31 or p105 in HPV-18. These oncogenes are transcribed as polycistronic transcripts and by alternative splicing, four E6 isoforms are generated: FLE6 (full-length E6), E6*I, E6*II and E6*X. The LCR region has domains for transcription factors binding as activator protein 1 (AP-1), Ying-yang 1 protein (YY1) and SP1 among others [30,31]. Specifically, AP-1 is a heterodimer composed by Fos and Jun family members able to bind a heptamer consensus sequence 5 ´-TGA(C/G) TCA-3 ´ into the LCR. AP-1 heterodimer is activated by p38, c- Jun N-terminal kinase (JNK), ERK1/2 and ERK5 Mitogen-Activated protein kinase (MAPK) pathways [32]. As the expression level and regulation of these transcription factors is cell-dependent, it is plausible that HPV-16 p97 promoter activity varies between tissues. In fact, using luciferase as a reporter, the p97 promoter activity was previously evaluated in different epithelial tumor and non-tumor cells transfected with LCR constructs. Cells from different tissues showed different activities of luciferase although unfortunately, bronchial or airway epithelial cells were not assessed and its relation with tobacco smoke was not previously evaluated [33]. In the present report we found a significant increase of E7 transcripts after incubation of SiHa cells with 10 μg/mL CSC. This increase was significantly higher compared with the activation of the HPV-16 LCR/p97 promoter in the same cells. Considering that expression of E6 and E7 by itself was able to increase the dose-response of BEAS-2B cells in the presence of CSC, it is possible to speculate some synergism between HPV oncoproteins and CSC for p97 promoter activation allowing an increase in E6/E7 transcripts.

On the other hand, in this study we found a significant increase in DNA damage such as DNA strands breaks (DSBs) after exposure to CSC of lung cells expressing HPV-16 E6 and E7 oncogenes (Figs 3 to 5). Both E6 and E7 were able to induce a significant DNA damage in lung cells exposed to 10 μg/mL CSC, however the effect of E7 was significantly higher. This is the first study reporting this effect in lung epithelial cells and suggesting collaboration for carcinogenesis. Previously, it was reported that E6 and E7 were able to increase oxidative DNA damage induced by CSC in cervical cancer cells [34]. Moreover, it has been reported that both E6 and E7 independently are able to induce DNA damage in different cells [35]. In fact, it was found that HPV-16 E6 disrupts the fidelity of DSB repair contributing to genetic instability in HPV associated tumors [36]. In addition, recently it was reported that E6* increases ROS levels and DNA damage in host cells [37]. On the other hand, Park JW et al suggested that E7 is associated to DNA damage at least in part through the inactivation of pocket proteins [38].

Previous studies used benzo[a]pyrene to show an interaction with HPV in cervical keratinocytes. In this respect, functional studies using organotypic “raft” cultures, have demonstrated that benzo[a]pyrene, depending on its concentration, is able to increase the number of virions and genomes of HPV [19]. While high concentrations of benzo[a]pyrene, favored virions synthesis, low concentrations of benzo[a]pyrene amplified the number of HPV genomes. The authors suggested that cyclin dependent kinase 1 (CDK1), activated after incubation with benzo[a]pyrene may be involved in virion maturation; a mechanism used by diverse viruses in morphogenesis [20]. In addition, the same authors suggested that an increased CDK1 activity could increase the viral persistence into the cells. In addition, it has been reported that benzo[a]pyrene increases the HPV E7 expression in a model of cervical raft cultures, and this overexpression is inhibited by curcumin, a potential chemopreventive agent. Because curcumin targets NF-κB and AP1 molecules, the authors suggested that a direct action of these molecules is involved in benzo[a]pyrene-induced E7 expression [18]. On the other hand, it was proposed that high-risk HPV E6 oncoprotein is able to activate serine/threonine-protein kinase CHK1 by phosphorylation at S345 in fibroblasts, in presence of genotoxic agents such as benzo[a]pyrene. As CHK1 is a kinase involved in G2 checkpoint, these findings suggested a mechanism for a synergism between a genotoxic agent and high-risk HPV E6 expression [39]. On the other hand, it was suggested that CHK2 is activated by phosphorylation by high-risk HPV for inducing the DNA damage response (DDR) under differentiation conditions [40]. Thus, HPV usurps the DDR machinery for efficient HPV replication. As benzopyrene or cigarette smoke condensate are able to induce gamma-H2X phosphorylation and DDR, is plausible a cooperation between both carcinogenic agents.

Recently, it was reported that tobacco smoke is able to induce early HPV transcription only when HPV persists as an episome [41]. In consequence, the authors of this study suggest that tobacco has a prominent role in early stages of HPV-related carcinogenesis. We cannot deny this possibility because we compare the activation of the p97 promoter with non-tumor cell lines (BEAS-2B and NL-20) and in the study of Wei et al, cell lines established from CIN (cervical intraepithelial neoplasia) where HPV persists as an episome were used. Future studies using cells from precursor lesions of lung cancer are warranted.

**Fig 6:**
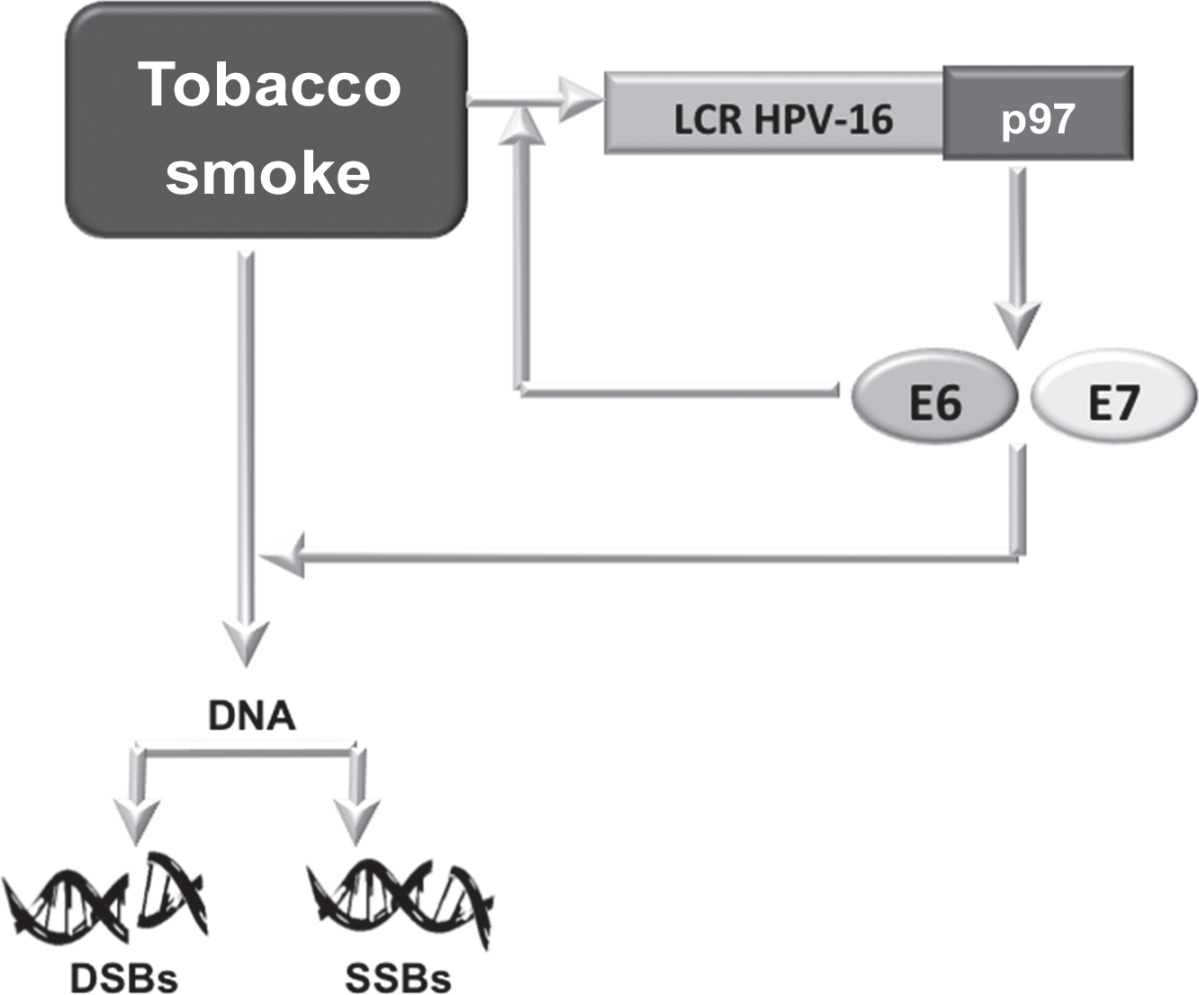
A model of interaction between CSC and HPV in lung cells is suggested. CSC is able to induce the activity of the p97 promoter in the context of high-risk HPV LCR conducting to E6 and E7 transcripts expression. The high-risk E6 and E7 oncoproteins sensitize lung cells for tobacco smoke-associated oxidative DNA damage and collaborate with tobacco smoke for p97 promoter activation.

Considering the findings presented in this study and others, a model of tobacco smoke and HPV interaction is proposed (Fig 6). In this model, tobacco smoke and HPV are able to collaborate at different levels: first, tobacco smoke induces the activity of p97 promoter in a dose-dependent manner with an intact LCR and the expressed HPV-16 E6 and E7 oncogenes sensitize lung cells for tobacco smoke-dependent oxidative DNA damage. In addition, HPV-16 E6 and E7 oncogenes are able to collaborate with tobacco smoke for p97 promoter activation in the context of non-tumor lung cells. More studies are warranted to analyze the clinical consequences of these findings including other cells or tissues that are potentially exposed to both HPV and tobacco smoke.

## Supporting Information

S1 Table Cell lines used in this study. (PDF)

S2 Table Primers used in this study. (PDF)

S1 Fig Schematic representation of the HPV-16 LCR obtained by PCR from SiHa cells. This region was inserted in pmiR-GLO vector for luciferase assays. Arrows indicate the sites that were used for cloning and restriction enzimes digestion. The PGK promoter was deleted and the backbone was used to obtain two new plasmids: one harboring the HPV-16 LCR/p97 region and another with the p97 promoter only region upstream of the firefly luciferase reporter gene.
(TIF)

S2 Fig Toxicity curves in cells used in this study exposed to different concentrations of CSC. Tumor and non-tumor lung cells where exposed to 0–100 μg/mL CSC and the viablity was maeasured after 96 hours of incubation using MTS assay.
(TIF)

S3 Fig Hydrogen peroxide exposition resembles to CSC for DNA damage in the presence of E6/E7 oncogenes. BEAS-2B (A) and A-549 (B) cells were exposed to 10 μM or 100 μM hydrogen peroxide for 0–60 min and DNA damage was evaluated using comet assay. The graphs are representative of three independent experiments (* = p<0.05; ** = p<0.01; *** = p<0.001, Total Area ± SEM).
(TIF)

S4 Fig γH2AX phosphorylation in lung cells exposed to CSC. BEAS-2B (A) and A-549 (B) cells were transfected with an empty vector (upper) or pLXSNE6/E7 oncogenes (below) and exposed to 10 μg/mL CSC. γH2AX phosphorylation was measured using immunofluorescence.
(TIF)

S5 Fig Dual HPV-16 E6/E7 silencing in BEAS-2B cells using different short-hairpins RNAs (shRNAs). Four shRNAs were transiently transfected in BEAS-2B lung cells ectopically expressing HPV-16 E6 and E7 oncoproteins. The expression of E6 and E7 transcripts was determined using RT-qPCR. The data were normalized using GAPDH expression.
(TIF)

S6 Fig P16 is overexpressed in BEAS-2B ectopically expressing HPV-16 E6 and E7 oncoproteins. BEAS-2B cells stably transfected with pLXSNE6E7 plasmids where pooled and p16 expression was assayed by Western blotting.
(TIF)
