# Supplementary material for: Tobacco Smoke Activates Human Papillomavirus 16 p97 Promoter and Cooperates with High-Risk E6/E7 for Oxidative DNA Damage in Lung Cells
Source: PLoS One. 2015 Apr 1;10(4):e0123029. doi: 10.1371/journal.pone.0123029 (PMC4382149; doi:10.1371/journal.pone.0123029)
Supplement: S1 Table — (PDF) [file pone.0123029.s001.pdf]

Region	Forward 5'-3'	Reverse 5'-3'	Amplicon (bp)	Function
LCR/P97	GAGGATCGAGATCTAGACCTAGATCAGTTTCC	ATGGCGCTGGGCCCTTCTTAATGTTTTTGGCATCTTCCATGGTGGCTTTACCAACAGTACCGGATTGCCAAGCTTTCCTGTGGGTCCTGAAACATTGC	1110	Cloning
p97	GAGGATCGAGATCTGGAACTTATATAATAATACTAAAC	ATGGCGCTGGGCCCTTCTTAATGTTTTTGGCATCTTCCATGGTGGCTTTACCAACAGTACCGGATTGCCAAGCTTTCCTGTGGGTCCTGAAACATTGC	234	Cloning
E7	CAATATTGTAATGG GCTCTGTCC	ATTTGCAACCAGAGACAACTGAT	120	qRT-PCR
GAPDH	CATGGTTCACACCCATGAGG	CGG GAAGCTTGTCATCAATGG	121	qRT-PCR
pmiR-GLO LCR/p97	CGTACAATTAAGGGATTATGG	GTAATGTCCACCTCGATATGTG	1320 and 444	Sequencing
