# Supplementary material for: Tobacco Smoke Activates Human Papillomavirus 16 p97 Promoter and Cooperates with High-Risk E6/E7 for Oxidative DNA Damage in Lung Cells
Source: PLoS One. 2015 Apr 1;10(4):e0123029. doi: 10.1371/journal.pone.0123029 (PMC4382149; doi:10.1371/journal.pone.0123029)
Supplement: S2 Table — (PDF) [file pone.0123029.s002.pdf]

Supplementary Table S2.

Cell line	Code	Origin	Phenotype
A-549	CCL-185	lung alveola	tumor
H-2170	CRL-5928	lung bronchial	tumor
NL-20	CRL-2503	lung alveola	non-tumor
BEAS-2B	CRL-9609	lung bronchia	non-tumor
SiHa	HTB-35	cervis uterine	tumor HPV-16
HeLa	CCL-2	cerxix uterine	tumor HPV-18
