# Supplementary figures and images for: Tobacco Smoke Activates Human Papillomavirus 16 p97 Promoter and Cooperates with High-Risk E6/E7 for Oxidative DNA Damage in Lung Cells
Source: PLoS One. 2015 Apr 1;10(4):e0123029. doi: 10.1371/journal.pone.0123029 (PMC4382149; doi:10.1371/journal.pone.0123029)

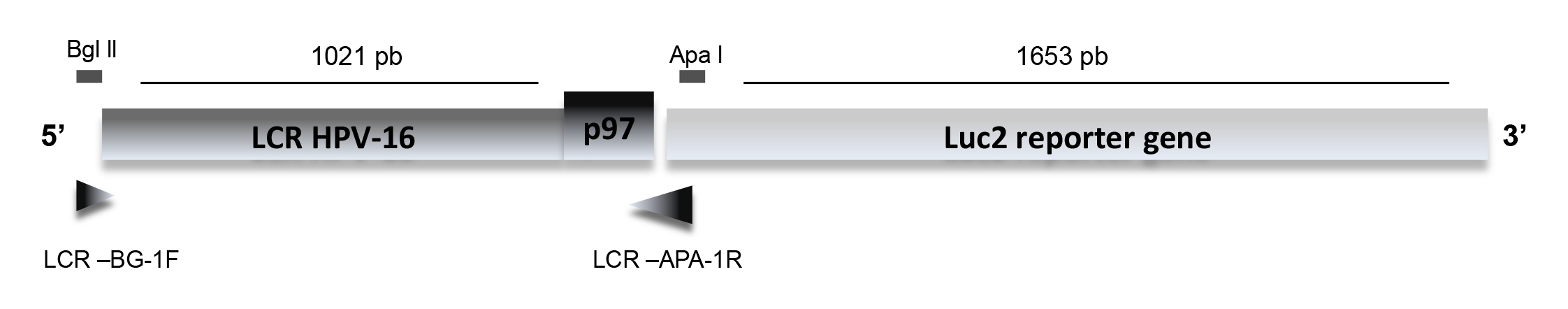

Supplement: S1 Fig — This region was inserted in pmiR-GLO vector for luciferase assays. Arrows indicate the sites that were used for cloning and restriction enzimes digestion. The PGK promoter was deleted and the backbone was used to obtain two new plasmids: one harboring the HPV-16 LCR/p97 region and another with the p97 promoter only region upstream of the firefly luciferase reporter gene. (TIF) [file pone.0123029.s003.tif]

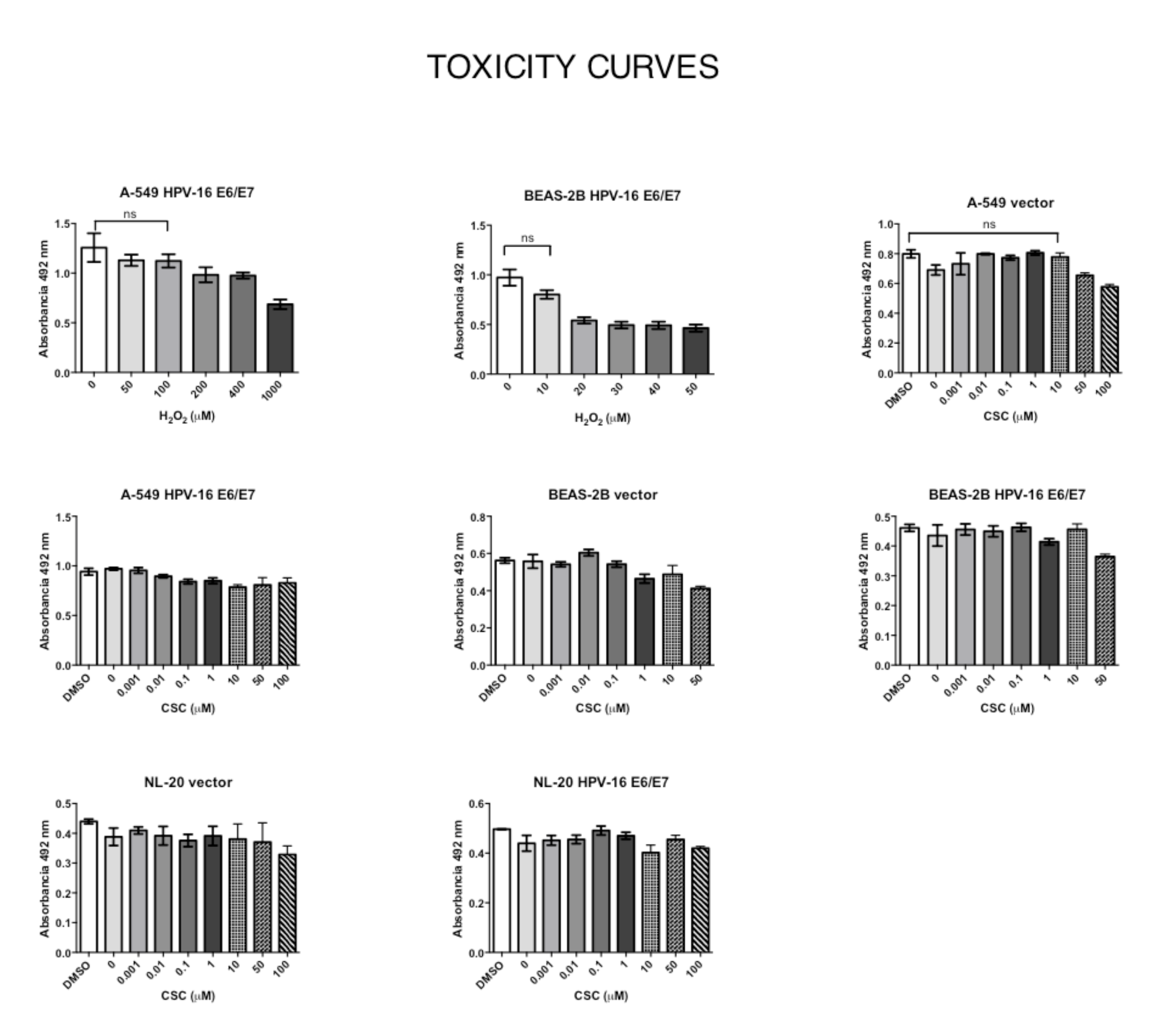

Supplement: S2 Fig — Tumor and non-tumor lung cells where exposed to 0–100 μg/mL CSC and the viablity was maeasured after 96 hours of incubation using MTS assay. (TIF) [file pone.0123029.s004.tif]

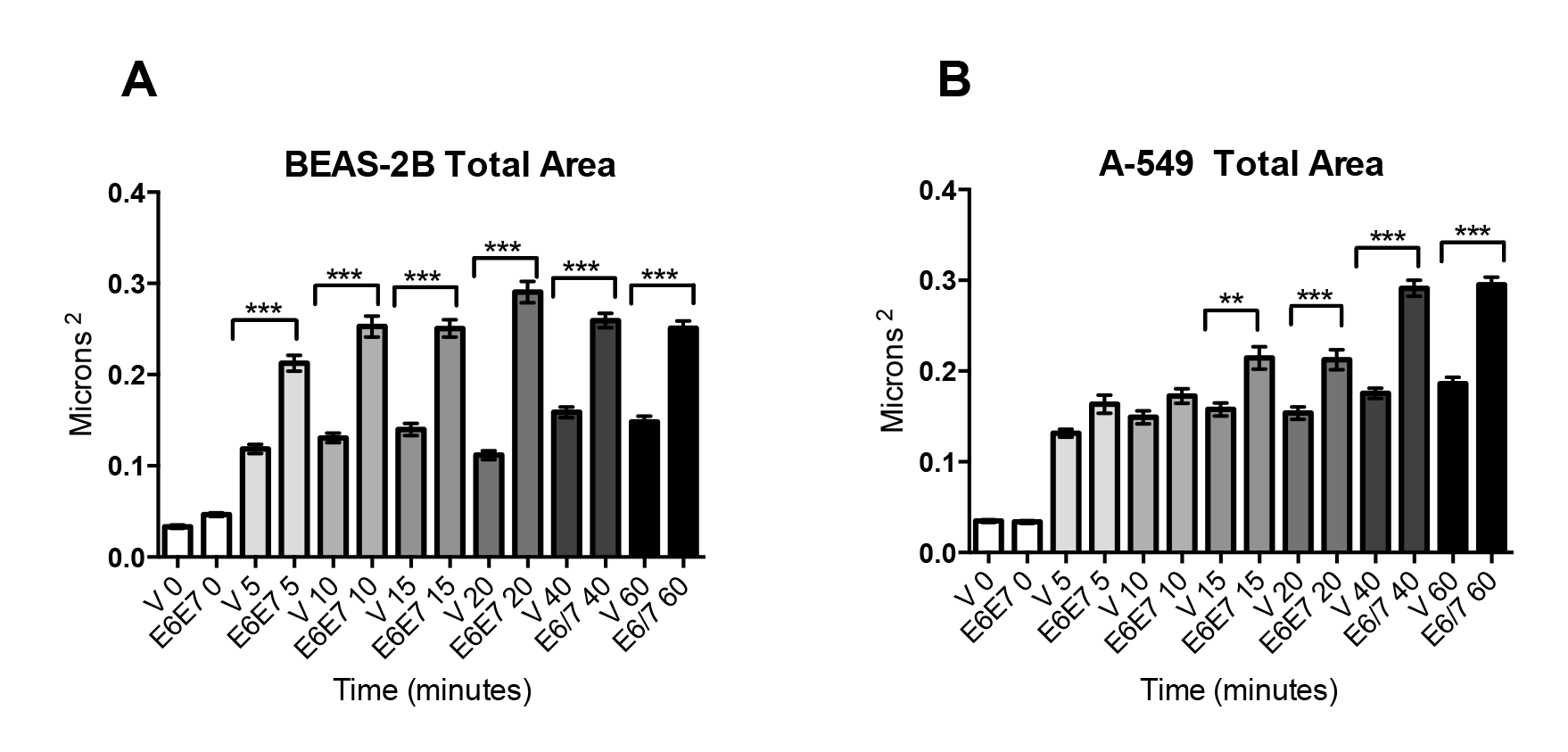

Supplement: S3 Fig — BEAS-2B (A) and A-549 (B) cells were exposed to 10 μM or 100 μM hydrogen peroxide for 0–60 min and DNA damage was evaluated using comet assay. The graphs are representative of three independent experiments (* = p<0.05; ** = p<0.01; *** = p<0.001, Total Area ± SEM). (TIF) [file pone.0123029.s005.tif]

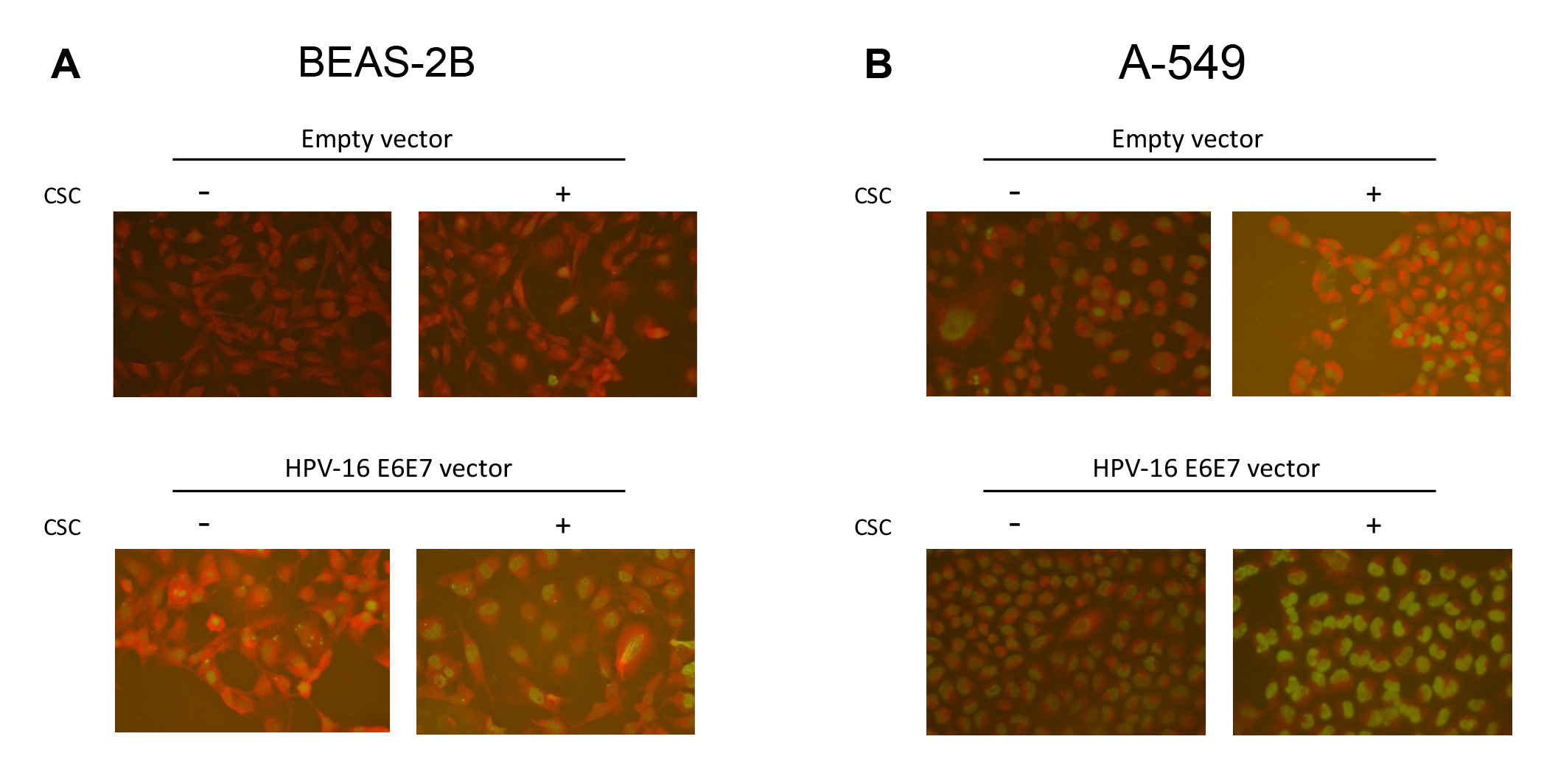

Supplement: S4 Fig — BEAS-2B (A) and A-549 (B) cells were transfected with an empty vector (upper) or pLXSNE6/E7 oncogenes (below) and exposed to 10 μg/mL CSC. γH2AX phosphorylation was measured using immunofluorescence. (TIF) [file pone.0123029.s006.tif]

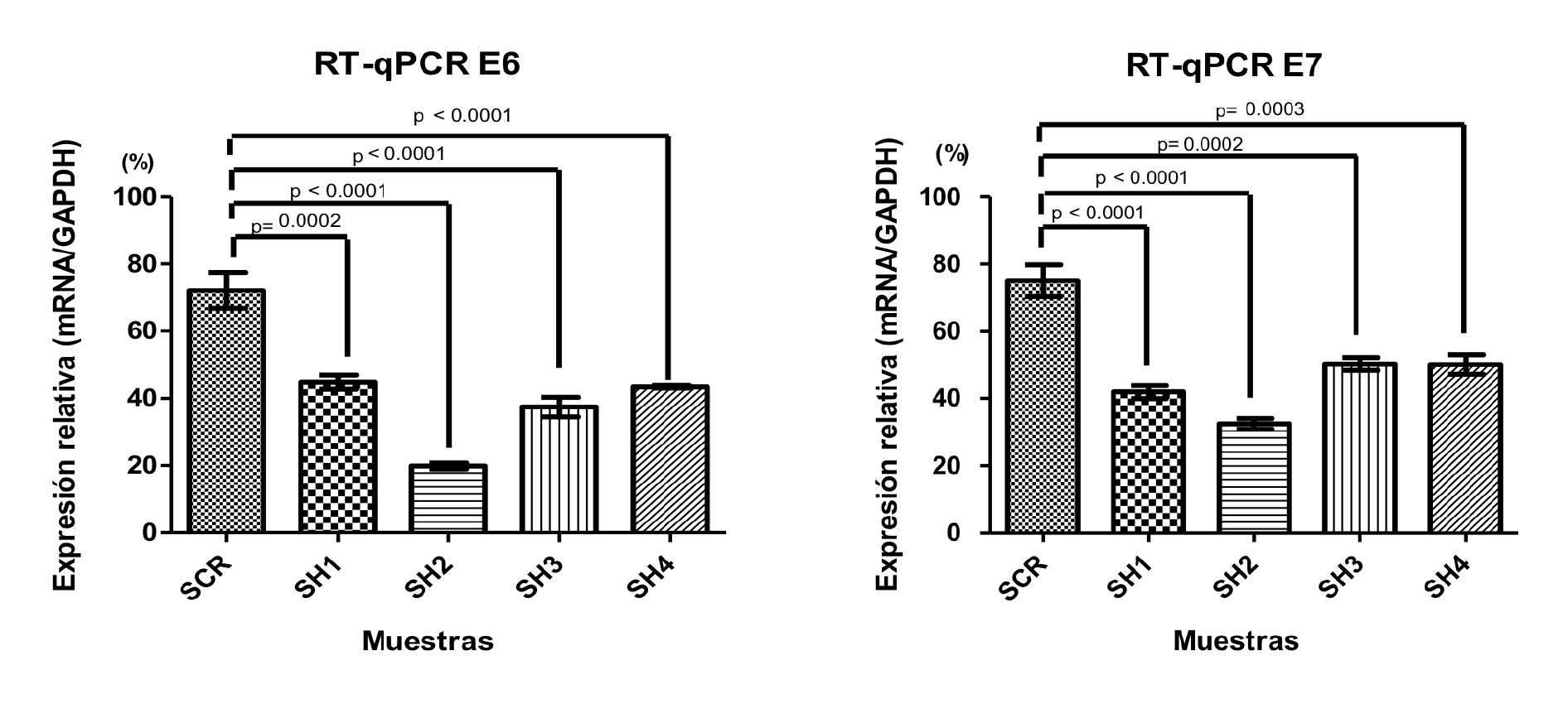

Supplement: S5 Fig — Four shRNAs were transiently transfected in BEAS-2B lung cells ectopically expressing HPV-16 E6 and E7 oncoproteins. The expression of E6 and E7 transcripts was determined using RT-qPCR. The data were normalized using GAPDH expression. (TIF) [file pone.0123029.s007.tif]

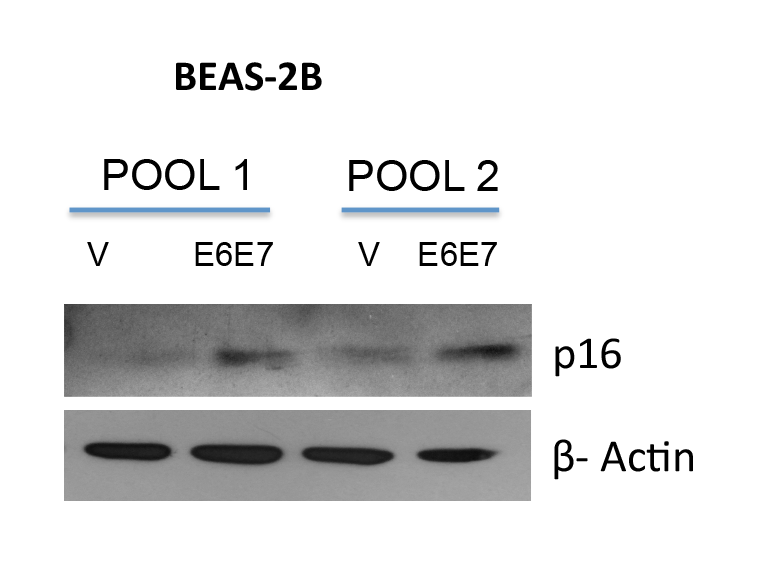

Supplement: S6 Fig — BEAS-2B cells stably transfected with pLXSNE6E7 plasmids where pooled and p16 expression was assayed by Western blotting. (TIF) [file pone.0123029.s008.tif]
